# ABT-888 enhances cytotoxic effects of temozolomide independent of MGMT status in serum free cultured glioma cells

**DOI:** 10.1186/s12967-015-0427-y

**Published:** 2015-02-26

**Authors:** Rutger K Balvers, Martine LM Lamfers, Jenneke J Kloezeman, Anne Kleijn, Lotte ME Berghauser Pont, Clemens MF Dirven, Sieger Leenstra

**Affiliations:** Brain Tumor Center; Department of Neurosurgery, Erasmus MC, Molewaterplein 50, Ee2236, 3015GE Rotterdam, The Netherlands; Department of Neurosurgery, St Elisabeth Hospital, Tilburg, The Netherlands

**Keywords:** Glioblastoma, PARP inhibition, GSC, Drug screening, Combination therapy, MGMT, Temozolomide, ABT-888

## Abstract

**Background:**

The current standard of care for Glioblastoma Multiforme (GBM) consists of fractionated focal irradiation with concomitant temozolomide (TMZ) chemotherapy. A promising strategy to increase the efficacy of TMZ is through interference with the DNA damage repair machinery, by poly(ADP-ribose) polymerase protein inhibition(PARPi). The objective of the present study was to investigate the therapeutic benefit of combination therapy in patient-derived glioma stem-like cells (GSC).

**Methods:**

Combination therapy feasibility was tested on established GBM cell lines U373 and T98. We developed an *in vitro* drug-screening assay based on GSC cultures derived from a panel of primary patient tissue samples (n = 20) to evaluate the effect of PARPi (ABT-888) monotherapy and combination therapy with TMZ. Therapeutic effect was assessed by viability, double stranded breaks, apoptosis and autophagy assays and longitudinal microscopic cell monitoring was performed. O-6-methylguanine-DNA methyltransferase (MGMT) status was determined by methylation assay and protein expression by western blots.

**Results:**

PARPi monotherapy was found to decrease viability by more than 25% in 4 of the 20 GSCs (20%) at 10 μM. TMZ monotherapy at 50 μM and 100 μM was effective in 12 and 14 of the 20 GSCs, respectively. TMZ resistance to 100 μM was found in 7 of 8 MGMT protein positive cultures. Potentiation of TMZ therapy through PARPi was found in 90% (n = 20) of GSCs, of which 6 were initially resistant and 7 were sensitive to TMZ monotherapy. Increased induction of double stranded breaks and apoptosis were noted in responsive GSCs. There was a trend noted, albeit statistically insignificant, of increased autophagy both in western blots and accumulation of autophagosomes.

**Conclusion:**

PARPi mediated potentiation of TMZ is independent of TMZ sensitivity and can override MGMT(-) mediated resistance when administered simultaneously. Response to combination therapy was associated with increased double strand breaks induction, and coincided by increased apoptosis and autophagy. PARPi addition potentiates TMZ treatment in primary GSCs. PARPi could potentially enhance the therapeutic efficacy of the standard of care in GBM.

## Introduction

Glioblastoma Multiforme (GBM) is the most common primary brain tumor for which there is no curative therapeutic option [1]. First-line treatment of GBM consists of tumor resection, followed by fractionated focal irradiation (IR) in combination with concomitant and adjuvant administration of the alkylating agent temozolomide (TMZ). Despite recent advances in understanding the molecular biology of GBM, the clinical perspective for newly diagnosed patients remains poor. The addition of TMZ to surgery followed by radiation has increased median survival from 12 to 15 months compared with IR monotherapy [2]. Hence, the need for therapeutic agents that can augment current treatment outcome is urgent.

It has been demonstrated that therapeutic efficacy of this chemo-radiation regimen, apart from clinical factors, depends on intrinsic molecular features of the tumor [3,4] such as methylation of the O6-methylguanine-DNA methyltransferase (MGMT) promoter gene and the subsequent lack of expression of the MGMT protein within GBM cells [5]. Several mechanisms have been identified that lead to the repair of alkylation-induced damage, such as base-excision repair (BER), mismatch repair (MMR) and homologous recombination (HR) repair [6,7]. The addition of DNA-repair system interfering agents may therefore potentiate the efficacy of TMZ treatment. Poly ADP Ribose Polymerase (PARP) proteins share the ability to transfer an ADP-ribose moïety from nicotinamide adenine dinucleotide (NAD^+^) to an acceptor protein and facilitate the accumulation of multiple sequential (poly) ADP-ribose units to the preceding one(s), a process termed PARylation. These proteins have been demonstrated to be essential in BER, after DNA damage induced by cytotoxic agents. PARP protein inhibitors (PARPi) were demonstrated to enhance therapeutic efficacy of several conventional cytotoxic agents, such as alkylating chemotherapy. One such agent, ABT-888 (*Veliparib®*), has been demonstrated to primarily bind to PARP-1and PARP-2, and inferiorly to PARP-3 and PARP-4 [8]. In humans, PARP-1 activity is responsible for 85-90% of PAR production with the remainder primarily attributable to PARP-2. Therefore, we will reference PARPi function from here on as diminished activity of PARP-1 and PARP-2.

Low passage glioma stem cell (GSC) cultures have been demonstrated to superiorly recapitulate genomic and gene-expression profiles, when compared with GBM cell lines and serum supplemented primary cultures [9,10]. The investigation of therapeutic effect of combined agents in patient derived material provides the ability to identify predictors of responsiveness *in vitro*. In the present study we investigated the sensitivity of a large panel of patient derived GSCs to monotherapy with both TMZ and PARPi. Additionally, we evaluated the ability of PARPi to potentiate TMZ cytotoxicity at varying clinically relevant doses. The ability of PARPi to overcome TMZ resistance is addressed in the context of MGMT expression in GSCs.

## Methods

### Cell cultures

All GSC primary cultures were derived from tumors of operated patients from the Erasmus Medical Center Rotterdam, The Netherlands. Prior to surgery, patients sign informed consent as approved by the Institutional Review Board of the Erasmus Medical Center Rotterdam. Tumor grading was performed by the local pathologist according to guidelines of the WHO grading of primary brain tumors. Freshly resected tumor samples were dissociated to establish primary cultures as described earlier [11]. Serum free medium constitutes DMEM-F12 with 1% penicillin/streptomycin, B27 (Invitrogen, Bleiswijk, The Netherlands), human EGF (5 ug/ml), human basic FGF (5 ug/ml) (both Tebu-Bio, Heerhugowaard, The Netherlands) and heparin (5 mg/ml) (Sigma-Aldrich, Zwijndrecht, The Netherlands). Tumor spheres were dissociated and passaged regularly for experiments or to derive material for characterization studies. T98 and U373MG conventional cell cultures were acquired from the American Type Culture Collection (ATCC, Manassas, VA) and propagated on DMEM supplemented with 10% fetal bovine serum and 1% penicillin + streptomycin.

### Viability and proliferation assays

Patient-derived tumor spheres were dissociated with Accutase™ (Invitrogen, Bleijswijk, The Netherlands), and cell lines T98 and U373 with trypsin. Cells were seeded at 1000 cells per well in 96 wells plates. For GSCs, wells were coated with extracellular matrix coating (BD Bioscience, Breda, The Netherlands). After 24 hours, cells were treated with TMZ (Sun Pharmaceutical Industries, Mumbai, India), or ABT-888 (Selleckchem, Huissen, The Netherlands), at indicated doses that have been demonstrated clinically relevant [12,13]. Combination therapy reagents were prepared by adding a mixed reagent in culture medium at equal volumes to monotherapy. Vehicle controls constituted of equal DMSO concentrations as needed for the dilution of TMZ therapy. After 5 days, viability was measured by performing the Cell Titer GLO™ assay according to the manufacturer’s instructions (Promega, Leiden, The Netherlands). All experiments were carried out in triplicate. T98 and U373 were used as positive and negative internal controls during GSC-based drug-screening experiments. Read-out was performed in a plate reader by measuring luminescence (Tecan Infinite 200, Tecan Benelux, Giessen, NL). Proliferation assays consisted of longitudinal imaging of cell confluency in the Incucyte HD Live Cell imaging incubator. Cells were seeded in 96 wellsplates and placed into the Incucyte incubator. Phase contrast images were acquired at 1-3hr intervals and confluence per well was calculated using a software platform supplied by the manufacturer (Essen Bioscience, Hertfordshire, UK).

Combination indexes of PARPi and TMZ combination therapy were obtained through Cell Titer GLO read-out, and calculated according to the Chou-Talalay procedure [14]. In short, serial dilution experiments with TMZ and ABT-888 monotherapy were carried out in order to determine the IC50 of each drug separately. Based on the Y-intercept and the slope of the curve derived from linearization of the viability data, the IC50 was calculated. Subsequently, ABT-888 and TMZ combination experiments were dosed by combining 3 stepwise derivatives (with 3 fold increases or decreases) of the IC50 value of both drugs in T98 and U373 respectively. Based on these experiments, standardized log derivatives of the dosages needed to elicit a decrease of viability (fraction affected, Fa) at a specific dose of each (combination of) agent(s) can be calculated. Subsequently the dose reduction index (DRI) of combination therapy is the ratio between the monotherapy dose and the combination therapy dose for a given Fa. The combination index is thus provided as the combined 1/DRI of both agents (1/DRI^tmz^ + 1/DRI^abt-888^), which should remain below 1 in order to demonstrate synergy.

### Double stranded breaks assays

To determine the induction of DSBs in GSCs, cells were seeded at a concentration of 2.5x10^4^ cells per well in matrigel coated 96 wells plates. Experiments were carried out as instructed by the manufacturer of the OxiSlect DNA Double Strand Break Staining Kit (Cell Bio Labs Inc, San Diego, CA, USA). In short, cells were incubated overnight and treated next day with 100 uM TMZ, 10 uM ABT-888 or combination therapy with both agents. Positive controls consisted of etoposide (0.1 mM) treated cells, whereas for negative controls we used non-treated controls. Readout was performed 16 hours post-treatment. Cells were fixed in 4%PFA/PBS, washed and stained according to manufacturer’s protocol with primary antiγH2Ax antibody solution (1:100) and counterstained with secondary FITC conjugated antibody solution (1:100). After washing, cells were incubated in the Incucyte and yH2Ax positive cells were counted per well with software as provided by the Incucyte manufacturer.

### Apoptosis and autophagy assays

To determine the induction of apoptosis or autophagy by mono- or combination-therapy GSCs were plated out in similar conditions with regard to culture conditions, drug dosing and preparations, and cell seeding, as described for the viability assays. The Cell Player Caspase 3/7 reagent (Essen Bioscience, Hertfordshire, UK) was added in a 1:1000 dilution to the culture medium at the time of treatment administration (according to manufacturers instructions). Read-out was performed after 48 hrs post-treatment by counting the ratio between apoptotic cells and total cells per well, using algorithms provided within the software of the Incucyte FLR.

For autophagy experiments, cells were seeded, treated and cultured as described above for previous *in vitro* experiments. To determine the induction of autophagy, we used the Cyto-ID Autophagy detection kit (Enzo Life Sciences, Raamsdonksveer, The Netherlands). This kit provides a monodansylcadaverine based dye that specifically stains autophagosomes [15]. To this end, cells were washed 16hrs post-treatment with assay buffer provided by the manufacturer. Next, cells were stained with the Cyto-ID green detection reagent for 30 minutes and subsequently washed twice more with assay buffer and cells were imaged in the Incucyte FLR for the detection of autophagosomes. Incucyte software was used to process imaging data. First a threshold was set for circumference and fluorescence intensity to identify autophagosomes. Next the autophagosomes per well were calculated using algorithms provided by the Incucyte manufacturer.

### MGMT methylation assay and western blotting

All samples used were derived from GS cultures passaged no more than 7 times. DNA and protein extraction was derived from fresh frozen pellets. Quantitative PCR on MGMT promoter methylation was assessed as previously decribed [16]. The following methylation specific primers were used F: TTTCGACGTTCGTAGGTTTTCGC and R: GCACTCTTCCGAAAACGAAACG. The un-methylated specific primers were F: TTTGTGTTTTGATGTTTGTAGGTTTTTGT and R: AACTCCACACTCTTCCAAAAACAAAACAQ. Western blots for MGMT protein expression were performed as follows. Samples were cultured, treated as indicated, and sequentially pelleted, washed twice in ice-cold PBS, and lysed in RIPA buffer with proteinase inhibitor (1%). Protein concentration was measured by performing the Bradford assay. The samples were diluted in Laemli buffer and run on 10% SDS-PAGE gel for electrophoresis. After running the gel, proteins were transferred to PVDF membranes in a BIO-RAD transfer system. Membranes were blocked with 5% milk in TBS-Tween (0.2%) and stained with primary antibodies at 4°C over night. After washing, secondary antibodies were applied for 1 hour with subsequent washing steps. Protein detection was achieved by using ECL chemiluminescent detection reagent (Pierce, Thermo Scientific Etten-Leur,NL). Antibodies used are anti-MGMT (Abcam, Cambridge MA, USA), LC3B (Cell Signaling, Danvers, MA, USA) and anti-β-actin (Clone C4, Millipore Amsterdam, The Netherlands).

### Statistical analysis

Data are presented as averages ± standard deviations as compared to non-treated controls. Statistical analysis was performed using the paired two-tailed Student’s *t*-test. Statistical significance was defined as p <0.05. Enhancement factors were determined as the ratio between the most effective single agent (between ABT-888 and TMZ) and the effect of combination therapy.

## Results

### PARP inhibition potentiates TMZ in sensitive and resistant conventional celllines

**Figure 1:**
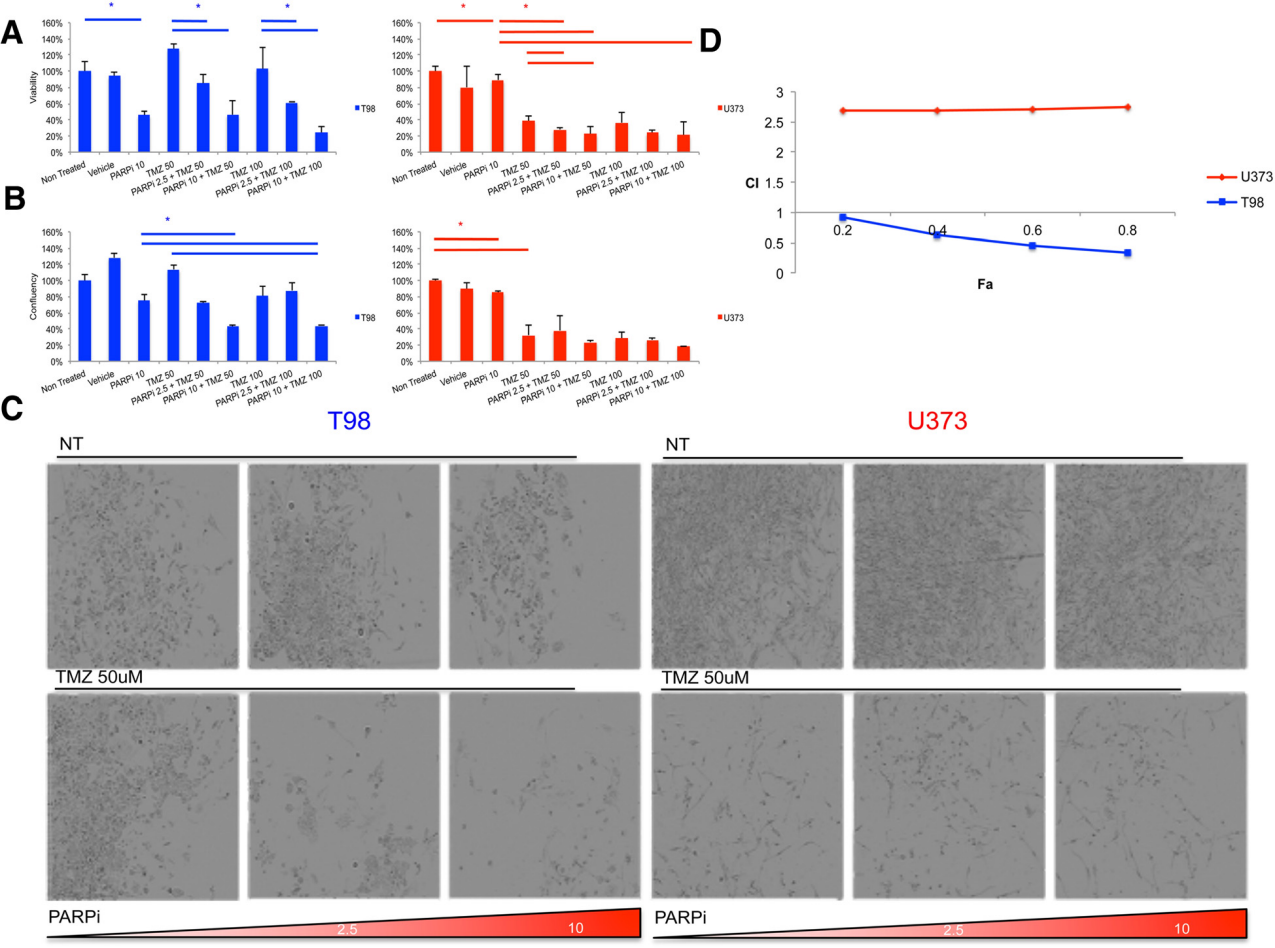
**PARPi potentiates TMZ therapy in conventional GBM cell lines on serum-supplemented medium. A)** Viability of T98 and U373 cell lines treated at the indicated doses with TMZ and PARPi. Viability is indicated as a measure of ATP conversion into luminescence. Lines above columns indicate significance between two conditions with a p-value <0.05. If monotherapy with TMZ is significant when compared to non-treated controls, the significance of combination therapy when compared to non-treated controls is related and therefore not defined as such in the figure. **B)** Viability as a measure of confluence per well as measured by microscopy. Read out is performed at 5 days post treatment. **C)** Microscopic images of U373 and T98. From left to right the PARPi dosage increases from 0 to 10 uM. Lower panel depicts combination therapy with TMZ at indicated dosages. Note the differential effect of PARPi monotherapy between T98 and U373. **D)** Combination Index (CI) plotted against Fraction affected (Fa) for T98 and U373. PARPi with TMZ combination therapy is synergistic in T98 but not in U373. CI < 1 demonstrates synergistic interaction of combination therapy with TMZ and PARPi at the indicated Fa. CI > 1 demonstrates antagonistic effect of combination therapy at the indicated Fa.

The addition of PARPi to TMZ treatment was tested in two conventionally used GBM cell lines, T98 and U373 respectively, for which the MGMT gene methylation and protein status have been previously reported [17]. Quantification of cytotoxicity was assessed by cell viability as well as monolayer confluency (Figure 1A and B). In accordance with previous reports [18], U373 was sensitive to TMZ monotherapy, whereas T98 was resistant (p>0.05 for both dosages compared to non**-**treated control). Conversely, T98 was sensitive to PARPi monotherapy whereas U373 was not. Comparative analysis of confluency and ATP-based viability generally correlated well. However, T98 susceptibility for ABT-888 monotherapy appeared more pronounced when assessed for viability than the confluency results suggest. Microscopic examination (Figure 1C) revealed fewer viable cells and drifting debris suggestive of cytotoxic effect of ABT-888 at 10 uM in T98 cells, which was not apparent in U373.

PARPi potentiated TMZ therapy in case of TMZ 50 μM in T98. Adding PARPi resulted in a dose dependent decrease of viability by 42% ±11% (2.5 μM PARPi) and 82% ±16% (10 μM PARPi). Similar results were found for the combination of TMZ at 100 μM with PARPi at 10 μM in T98 (p<0.001). In U373, combination treatment of TMZ 50 μM with 2.5 uM PARPi was more effective than mono-treatment, however, the net decrease was small (10% ±2.6%, p<0.05), while not significant for TMZ 100 μM/10 uM PARPi.

To evaluate synergy between the two agents in a systematic manner, the Chou**-**Talalay combination index (CI) was determined for both agents in these two cell-lines [14]. T98 was comparatively resistant to TMZ with an IC50 at 5 days of 415.5 μM (R^2^ = 0.97), while the IC50 of U373 was 66.8 uM (R^2^ = 0.94) at 5 days. T98 was relatively sensitive to PARPi with an IC50 of 16.46 μM (R^2^ = 0.98) compared to an IC50 of 58.6 uM (R^2^ = 0.97) for U373. Next, the combination index (the ratio between dose reduction of combined treatment and monotherapy with either agents individually) was plotted. For T98 the combination of PARPi and TMZ was found synergistic with a CI below 1 for all Fa (fraction affected) (Figure 1D). In contrast, the CI U373 consistently demonstrated a CI>1, suggestive of antagonized cytotoxicity of ABT-888 when combined with TMZ in this cell line.

### PARPi and TMZ monotherapy screening experiments in GSCs

**Figure 2:**
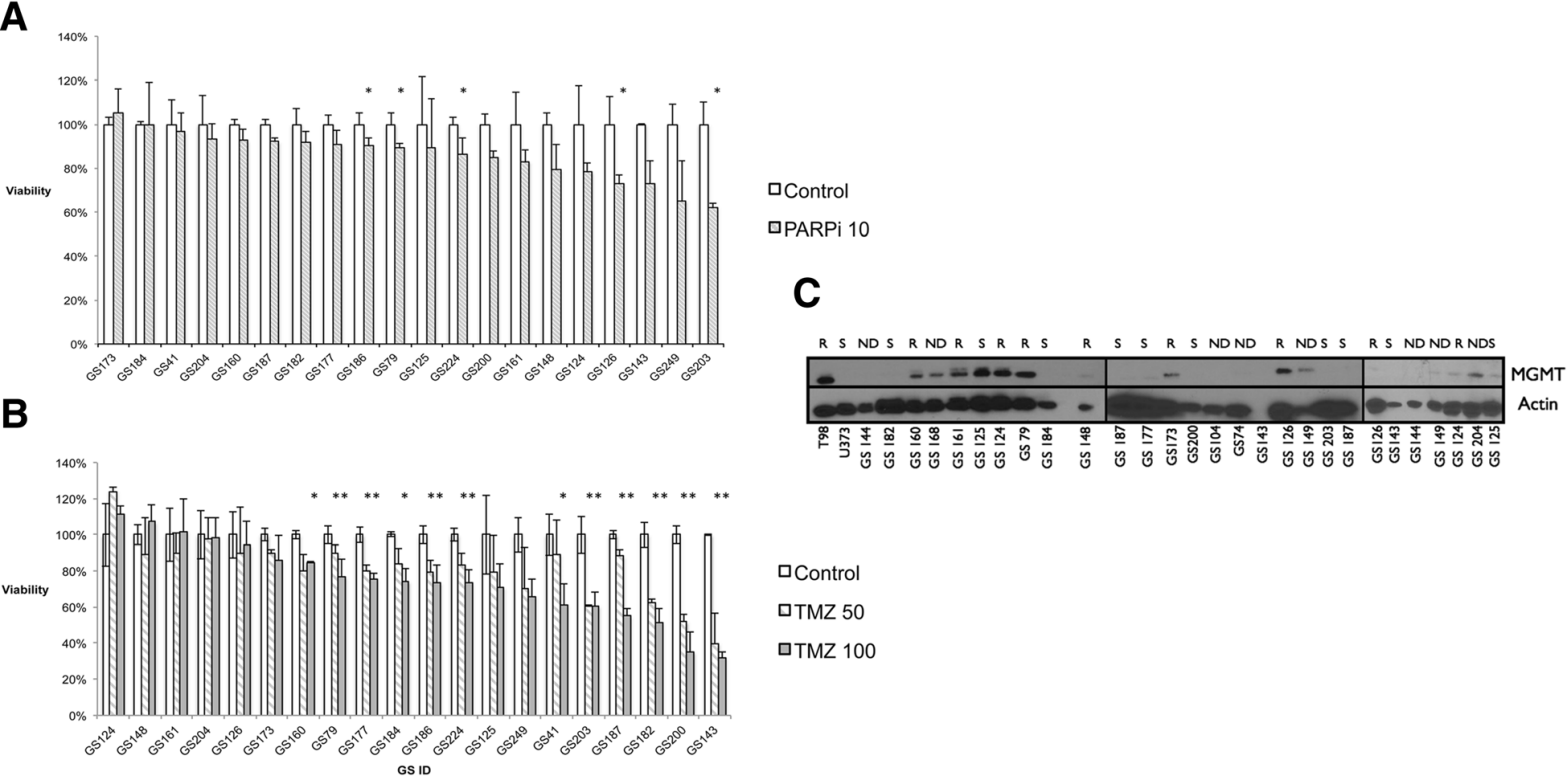
**PARPi and TMZ monotherapy efficacy in GS cultures. A)** Panel of GS cultures tested for sensitivity to PARPi at 10 uM. Readout of viability was performed at day 5 and is depicted as a percentage when compared to non-treated controls. * Illustrates a p-value <0.05. **B)** GS culture panel tested against indicated concentrations in uM of TMZ monotherapy. Viability at day 5 is indicated as percentages when compared to paired non-treated controls. * indicates p < 0.05 as compared to non treated controls. **C)** Overview of western blotting results derived from GS cultures. MGMT protein expression is indicated with actin protein expression used as a loading control. TMZ sensitivity is indicated with an S (sensitive), R (resistant) and ND (TMZ sensitivity not determined in monotherapy screen). For several GSCs protein isolates were loaded in separate runs as technical controls.

Molecular characteristics as found in primary GBM tissue, are inferiorly recapitulated in conventional cell lines such as T98 and U373, when compared to early passages of serum-free patient-derived cultures [10,19]. Therefore, we tested 20 GSC cultures from high-grade malignant glioma for the PARPi ABT-888 and TMZ sensitivity (overview of clinically relevant information in Table 1). GSC cultures were labeled sensitive if more than 25% reduction in viability was measured as compared to non-treated controls. For PARPi monotherapy at 10μM, this was found in 4/20 cultures (20%), (Figure 2A).

**Table 1:** **Overview of GSC panel drug screening results**

**GS ID**	**Histology**	**Age**	**OS**	**MGMT Blot/Methyl**	**PARPi**	**TMZ**	**Enhancement factor TMZ 50**	**p-value**	**Enhancement factor TMZ 100**	**p-value**
GS249	GBM	53	215	- / ND	0.30	0.66	3.06	0.01	2.92	0.03
GS187	GBM	73	394	- / UM	0.12	0.55	2.22	0.00	2.31	0
GS41	AOD	64	1316	ND	0.11	0.61	1.74	0.08	0.92	0.19
GS125	GBM	75	234	+ / UM	0.21	0.71	1.59	0.17	1.78	0.06
GS203	GBM	64	554	- / M	0.39	0.61	1.68	0.01	1.71	0.01
GS184	GBM	50	833	- / M	0.16	0.74	1.40	0.10	1.66	0.01
GS186	GBM	49	792	- / M	0.21	0.74	1.30	0.03	1.49	0
GS224	GBM	58	101	- / M	0.17	0.73	1.15	0.20	1.20	0.03
GS177	GBM	63	843	- / M	0.20	0.76	1.14	0.01	1.15	0.2
GS182	GBM	70	835	- / M	0.38	0.51	1.14	0.02	1.31	0.05
GS200	GBM	57	321	- / ND	0.48	0.35	1.16	0.02	1.18	0.08
GS143	GBM	54	395	- / ND	0.60	0.32	1.17	0.40	1.32	0.09
GS126	GBM	41	962	+ / M	0.27	0.06	1.68	0.02	1.69	0.01
GS79	GBM	71	384	+ / UM	0.11	0.23	2.11	0.00	2.48	0
GS124	GBM	71	1033	+ / ND	0.22	−0.11	1.19	0.40	1.19	0.06
GS148	AOD	82	97	+ / UM	0.20	−0.07	1.15	0.80	1.15	0.06
GS173	GBM	68	138	+ / ND	−0.05	0.14	1.24	0.07	1.73	0.01
GS204	GBM	50	18	+ / UM	0.07	0.02	1.17	0.03	1.17	0.04
GS161	GBM	62	264	+ / M	0.17	−0.02	0.80	0.04	0.80	0.2
GS160	GBM	46	46	+ / UM	0.07	0.16	0.83	0.03	0.75	0

The separation between TMZ sensitive and resistant groups is based on monotherapy results for TMZ 100 μM results. Significance is depicted as a p-value derived Student’s *T*-test of the most effective single agent compared to combination therapy. For OS, boxes without shading depict patients that were alive at the moment of composing this article. Abbreviations as followed; AOD = Anaplastic Oligodendroglioma, GBM = Glioblastoma Multiforme, OS = Overall Survival in days, MGMT = O6-methylguanine-DNA methyltransferase, Blot = Western blotting results which can be positive (+) or negatieve (−), Methyl = Methylation assay which can be Methylated or UnMethylated). ND = Not determined.

Sensitivity to TMZ was tested at two standard concentrations of 50 μM (low dose) and 100 μM (high dose). These concentrations are derived from experiments with T98 and U373 as the optimal concentrations to detect additional effect by combination therapy in both TMZ sensitive and resistant cultures, within physiological ranges as found in plasma and CSF of patients treated with TMZ [20]. Low dose TMZ induced more than 25% viability loss in 5/20 cultures (25%) (Figure 2B). High dose TMZ treatment had therapeutic effect in 12/20 cultures (55%). Based on these results we categorized the 20 GSC cultures into PARPi and TMZ resistant and sensitive cultures based on the initial monotherapy screen (Table 1). Six of the 20 cultures were tested at least three times for reproducibility of the results, which demonstrated consistent TMZ and PARPi therapeutic efficacy profiles over multiple passages (data not shown).

Since therapeutic response of malignant glioma to TMZ is known to be highly correlated with MGMT promoter methylation and a subsequent decrease in MGMT protein expression [5,21], we performed Western blotting of MGMT protein in 19 out 20 cell cultures tested in our initial drug screening (Figure 2C). MGMT was expressed in 9 GSC cultures (MGMT(+)), of which 8 were found to be resistant to 100 μM of TMZ. Conversely, all 11 MGMT negative (MGMT(-)) cultures were found sensitive to TMZ therapy. Together, TMZ sensitivity was highly correlated to MGMT expression (p=0.007). In addition we determined the promotor methylation status, which generally correlated well with protein expression (n = 11/14, 78%). Interestingly, the sensitivity to ABT-888 was also correlated to MGMT expression, since MGMT expressing GSCs were significantly more resistant to ABT-888 10 uM monotherapy compared to MGMT negative GSCs (p=0.01).

### PARPi addition potentiates TMZ treatment in primary GSCs

To determine the ability of the PARPi ABT-888 to potentiate TMZ efficacy, we evaluated the therapeutic effect of combination therapy in the aforementioned GSC cultures (n=20). Enhancement factors were calculated as the ratio between the most effective drug in monotherapy and the effect of combination therapy. Efficacy enhancement by combination therapy was determined as an enhancement factor >1. As shown in Table 1, enhancement of 50 μM TMZ by10 uM PARPi was found in 18 of 20 cultures (90%) of which 10 cultures had a p-value of <0.05. Average and median decrease of viability as compared to TMZ monotherapy was 22.8% and 21.9%, respectively. For high dose TMZ monotherapy, 17 out of 20 (65%) cases were potentiated of which 11 had a p-value <0.05. In this group the average and median reduction in viability was 23% and 28.8% (Table 1). Except for GS41, enrichment factors between high and low dose TMZ were similar or increased with the higher dosing of TMZ, suggesting a dose response effect of combination therapy. In conclusion, 18 out of 20 GSC cultures were coined responders to combination therapy (R), and two MGMT(+) cultures were coined non-responders (NR).

We found that PARPi potentiated four of the MGMT(+) GSC cultures (p<0.05); at both low and high dose TMZ. Similarly, in 4 out of 8 MGMT negative cultures we discovered additive effect of combination therapy (p<0.05). Student’s *t*-test revealed no significant difference between MGMT(+) or MGMT(-) GSCs with regard to ABT-888 potentiation of TMZ-therapy (p=0.71 low dose, and p=0.50 high dose respectively). Similarly, TMZ sensitivity as determined by monotherapy screening was not correlated to ABT-888 potentiation (p=0.24 low dose TMZ, p=0.42 high dose TMZ, respectively). In the single culture (GS173) for which the lack of MGMT expression was not correlated to TMZ sensitivity, we found PARPi mediated potentiation of TMZ as well. Thus, PARPi can potentiate TMZ efficacy regardless of the sensitivity to TMZ or MGMT status.

### PARP inhibition increases TMZ induced double stranded breaks, autophagy and apoptosis in GSCs

**Figure 3:**
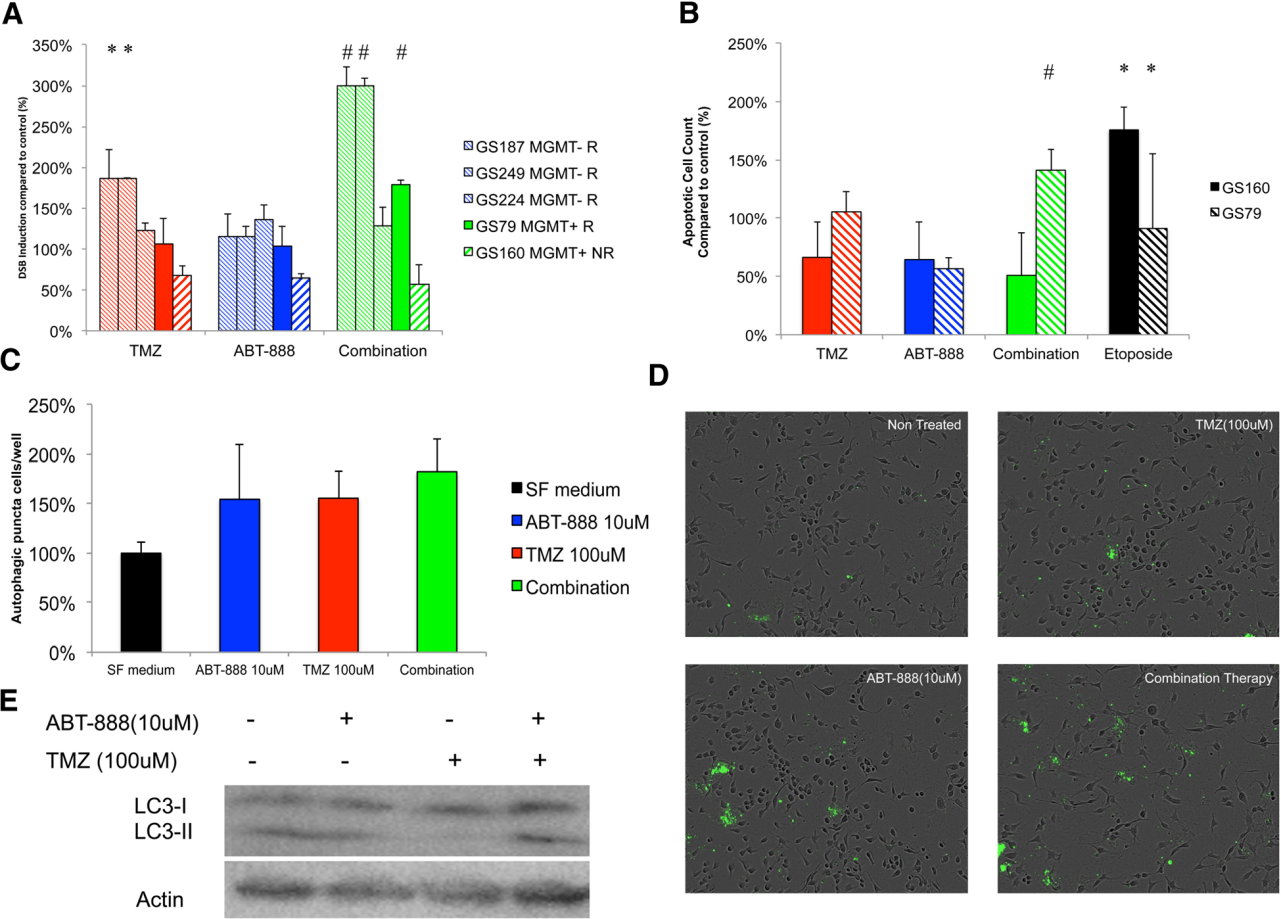
**PARP inhibition increases TMZ induced double stranded breaks, autophagy and apoptosis in GSCs. A)** DSB induction is significantly enhanced by combination therapy. GSCs, MGMT(+) and MGMT(-) were scored for γH2Ax positive cells, as an indicator for DSB induction,16 hrs post-treatment. GS160 results are all significant as compared to non-treated controls. For the other cultures, significance is indicated as described in the following; * indicates p < 0,05 as compared to non-treated controls. # indicates p < 0.05 as compared to monotherapy. R = combination therapy responder, NR = non-responder. **B)** Apoptosis induction is significantly enhanced by combination therapy in responder GSCs but not in non-responders. Wells were scored for apoptosis positive cells by live imaging. Read out was performed at 48 hrs post-treatment. Apoptotic cells were significantly (p < 0.05) increased after combination therapy as compared to monontherapy in GSC79. For GS160 cells were not affected by TMZ/ABT-888 combination therapy while the positive control (etoposide 0.1 mM) was successfully inducing apoptosis. **C)** TMZ and ABT-888 induce autophagy in GS79, which is augmented by combination therapy. Autophagosomes were counted per well and compared to non-treated controls. Readout was performed 16 hrs post-treatment. Compared to controls, all treatment conditions were significantly increased p < 0.05. However, combination therapy vs. monotherapy with ABT-888 or TMZ was found not significantly increased (p = 0.3 and p = 0.4). **D)** Representative images acquired by the Incucyte FLR of GS79 stained for autophagosomes at indicated treatment conditions. Note the accumulation of speckled dots in treated cells, specifically localized in enlarged cytopathic cells. **E)** Combination therapy enhances autophagic flux as compared to monotherapy in GS79. Western blot 24 hrs post-treatment indicates the accumulation of LC3-II (lipidated isoform) that is indicative for increased autophagic flux. Actin blots serve as loading controls.

The progression of alkylation based single stranded breaks (SSBs) to double stranded breaks (DSBs) has been proposed to be fatal in cancer cells. While MGMT can facilitate repair of SSBs, the rationale behind the addition of ABT-888 to TMZ is to comprise the DNA-repair cascade needed to prevent the induction of DSBs after alkylation induced SSB formation. To address this proposed role of DSB induction as an outcome of therapeutic efficacy in our GSC panel, three MGMT(-) and two MGMT(+) GSCs were tested for DSB induction when treated with TMZ, ABT-888 or combination therapy. The induction of DSBs by TMZ monotherapy was significant (p < 0.01) in 2 out of 3 MGMT(-) GSCs (Figure 3A). Monotherapy with ABT-888 did not yield a significant increase in DSB induction. Interestingly, DSB induction was not apparent in one non-responder GS160 (MGMT(+)), suggestive of the premise that DSB induction is needed for combination therapy to potentiate TMZ-monotherapy.

Next we assessed the induction of apoptosis and autophagy, two pathways implicated to be upregulated in response to both TMZ and PARP inhibitor treatment [22-24]. We found that, in GS79 (MGMT(+), R), apoptosis was significantly (p<0.05) increased by combination therapy as compared to monotherapy (Figure 3B). The resistance to apoptosis induction following drug administration in GS160 further substantiated the hypothesis that DSB induction is mandatory for cell death after combination therapy. Autophagy has been implicated both in the induction of senescence after cytotoxic insult to facilitate DNA repair, as well as the induction of caspase dependent cell death (apoptosis). The induction of autophagy by therapeutic agents can be monitored through the accumulation of autophagic vacuoles by fluorescent microscopy or the conversion of LC3B-I into the lipidated LC3B-II [25]. We found that both TMZ and ABT-888 induced autophagy in GS79 (Figure 3C) through a fluorescence based autophagy induction monitoring kit. In addition, combination therapy was found that have an additive effect with regard to the induction of autophagosomes on microscopic imaging (Figure 3C-D). Furthermore, the conversion of LC3B-I to LC3B-II, a golden standard for the assessment of autophagic flux [26], demonstrated to be increased in combination therapy on western blot (Figure 3E).

## Discussion

We here report the potential of ABT-888 as an additive to TMZ chemotherapy for the treatment of GBM. ABT-888 is a selective inhibitor of both PARP-1 and PARP-2 protein function, which are together responsible for the majority of PAR activity [8]. The results presented here, demonstrate that PARPi decreases proliferation and viability of the glioma cell line T98 and of a substantial subset of patient-derived GSCs. Furthermore, combined treatment with PARPi and TMZ leads to significant potentiation of therapeutic efficacy in the majority of GSCs, irrespective of MGMT protein expression. In addition, we found that MGMT+ GSCs are also significantly more resistant to ABT-888 monotherapy, as compared to MGMT(-) GSCs. We have found evidence that the potentiation of TMZ therapy by ABT-888 is dependent on the induction of DSBs, which coincides with increased apoptosis and autophagy in responsive cells.

Several studies have demonstrated the feasibility of PARPi as an adjuvant in the treatment of malignancies [27]. PARP activity has been implicated in both single stranded break (SSB) repair and double stranded break (DSB) repair [28]. Indeed, PARPi increased the efficacy of both TMZ and other alkylating agents in preclinical models of GBM and other solid tumors [29,30]. Although previous studies have demonstrated the feasibility of this strategy in preclinical models based on serum supplemented conventional cell lines [29,31,32], we here address this form of combination therapy in a panel of patient-derived GSCs. This is important since conventional cell lines have been demonstrated to poorly mimic the genomic and transcriptomic landscape of GBM [19]. In addition, GSCs have been postulated to play an important role in therapy resistance to current standard of care for patients [33-36]. The results for TMZ monotherapy in our GSC panel are in congruence with previous publications [37-40] and reproduce MGMT protein expression and subsequent TMZ resistance as found in parental tumors [41]. Therefore, this study also confirms that GSC panels are a suitable platform for assessing the potential of TMZ combination therapy with other promising agents.

Although it is expected for most GBM samples to be resistant to monotherapy with PARPi, since most GBM have intact DNA-repair-signaling pathways, testing for ABT-888 monotherapy effect demonstrated that a substantial (20%) part of our panel was sensitive to clinically relevant dosages of ABT-888. This has not been demonstrated in patient derived glioma samples with ABT-888 before, although other PARPi have had similar effects in conventional cell lines [32]. Therefore, we conclude that PARPi could be beneficial to therapeutic efficacy in GBM, apart from interacting synergistically with TMZ. Previous studies have demonstrated phosphatase and tensin homologue (PTEN) deficient cells to be particularly vulnerable to PARPi monotherapy through compromised homologous recombination repair [7]. For T98, susceptibility to PARPi could be related to a mutation in the PTEN gene, as reported for this cell line [42]. In GBM tissue profiling studies, PTEN signaling has been demonstrated to be dysfunctional in 40-65% of GBM cases [43,44]. However, other genes have also been implicated in the efficacy of PARPi *in vitro* as well, such as BRCA-1/2 or CDK5 [45,46]. Future research on PARPi therapy may therefore benefit from focused research on the identification of predictive molecular signatures for the susceptibility to PARPi mono- or combination therapy in malignant glioma.

Our study shows that PARPi enhances TMZ chemotherapy in 18 out of 20 cultures at varying doses (90%). The potentiating effect of PARPi to TMZ is found in both TMZ resistant and sensitive cultures. Combination therapy was effective in 6 out of 8 MGMT expressing GSC cultures, suggesting that the additive effect is independent of MGMT status. At the time of finalizing this manuscript, Tentori and colleagues, published a similar study that demonstrated synergistic effect of combined PARPi (inhibitor GPI-15427) and TMZ therapy in a panel of 10 GSCs, data which are largely analogous with our findings [47]. These results indicate that PARPi has the potential to improve therapeutic efficacy of TMZ in both responders and non-responders to TMZ monotherapy.

Since the current chemo-irradiation treatment regimen has considerable side-effects, the possible potentiation of TMZ by PARPi may also be considered in the context of TMZ dose reduction, leading to reduced side-effects, and prolonged therapeutic dosing in patients undergoing chemo-irradiation. As combination therapy efficacy may be unrelated to MGMT status, this may provide for an interesting alternative to patients with a recurrence after TMZ treatment. As such, other alkylating agents (e.g CCNU in the context of PCV, which is frequently used for recurrent oligodendroglioma) may be of considerable interest to study in combination with PARPi [48].

The mechanisms through which PARPi enhances TMZ induced cytotoxicity may be ambiguous. For one, after the induction of SSBs by TMZ, PARPi prevents the binding of PARP1-2 to damaged DNA foci to facilitate BER. The PARPi mediated prevention of BER enhances the formation of DSBs, which would eventually be lethal. Our DSB and apoptosis induction assays provide compelling evidence that this hypothesis holds truth in combination therapy responder GSCs. Next to that, the induction of autophagy after TMZ or ABT-888 might provide an alternative mode of programmed cell death that is induced after combination therapy. Whereas monotherapy might induce autophagy as a mode of cellular adaptation to the cytotoxic event, the cumulative stress elicited by combination therapy might induce autophagic cell death, which has been described as a context dependent mode of programmed cell death induced after a tipping point of autophagic flux is reached [49].

## Conclusion

This study underscores the potential of PARPi as an enhancer of TMZ treatment of patients with GBM. Furthermore, in select cases PARPi could elicit therapeutic effect as a single agent. We have demonstrated the efficacy of PARPi in GSCs, which are a more relevant *in vitro* model for GBM than the conventional cell lines used previously. PARPi-mediated potentiation of TMZ therapy is independent of TMZ monotherapy sensitivity or MGMT protein expression, which makes PARPi a promising drug for addition to the current standard of care chemo-irradiation regime.
